# Effects of Muscular Fatigue on Position Sense in Two Phases of the Menstrual Cycle

**DOI:** 10.3390/jfmk9030115

**Published:** 2024-06-29

**Authors:** Elmina-Eleftheria Roditi, Themistoklis Tsatalas, Giorgos K. Sakkas, Yiannis Koutedakis, Giannis Giakas, Christina Karatzaferi

**Affiliations:** 1 Experimental Physiology—CREHP, Department PE & Sports Science, University of Thessaly, Karyes, 42100 Trikala, Greece; elminion@yahoo.com (E.-E.R.); y.koutedakis@uth.gr (Y.K.); 2 Biomechanics and Ergonomics—CREHP, Department PE & Sports Science, University of Thessaly, Karyes, 42100 Trikala, Greece; ttsatalas@uth.gr (T.T.); ggiakas@uth.gr (G.G.); 3 Lifestyle Medicine—CREHP, Department PE & Sports Science, University of Thessaly, Karyes, 42100 Trikala, Greece; 4 School of Sports and Health Sciences, Cardiff Metropolitan University, Cardiff CF5 2YB, UK; 5 Faculty of Education, Health and Wellbeing, University of Wolverhampton, Wolverhampton WV1 1LY, UK

**Keywords:** proprioception, knee joint position sense, neuromuscular fatigue, women athletes

## Abstract

It is generally accepted that local muscular fatigue can negatively affect position sense. Interestingly, it has been proposed that in women, position sense and neuromuscular coordination may be affected by fluctuations of estrogen and progesterone levels. The aim of this study was to examine the possible effect of localized muscle fatigue on knee joint position sense at two phases of the menses: follicular and luteal. Twenty physically active females aged 19–30 years, with normal menses, volunteered for this study. An isokinetic dynamometer was used to evaluate proprioception and perform the fatigue protocol of the knee extensors and flexors. Knee proprioception at rest and after fatigue at three knee target angles (30°, 45°, 60°) was measured. A three-way ANOVA analysis with repeated measures was performed. The results showed that the main effect of fatigue was significant, but no main effect of the menstrual cycle phase was found. Additionally, a main effect was found for the target angle (more flexed target knee joint angles were associated with larger angular error deviations). In conclusion, localized muscle fatigue can significantly reduce the accuracy of active knee joint repositioning in both the luteal and the follicular menstrual phases in young, physically active healthy women.

## 1. Introduction

Proprioception is an important part of neuromuscular performance and postural control, and can be defined as an individual’s awareness of their extremities’ position and motion in space [1]. The proprioceptive system is complex and encompasses both spinal and cortical projections and reflective pathways [2,3]. Proprioception can be divided into two parts: the sense of joint position and the sense of limb movement (kinaesthesia) [1]. Position sense and kinaesthesia are abilities that help control the normal execution, range, and frequency of movement, the position of joints, and finally muscle and ligament stretching while helping to avoid injuries [4].

Proprioception can be quantified by different tests. The most frequently used tests are joint position sense threshold tests in which subjects are asked to detect passive movement, and balance tests [3,5,6]. Some tests can also examine one’s ability to actively return a limb to a target joint angle, i.e., active repositioning [7], which can be thought of as more relevant to field applications.

Generally, it is accepted that local muscular fatigue can negatively affect proprioception [8]. Also, the joint position sense seems affected by muscle damage due to eccentric exercise [7]. Proprioception may also be affected by factors such as age, gender, and other [9,10], but its mechanisms have not yet been clarified.

Proprioception is critical for injury prevention as it allows correct technique execution but also facilitates swift adjustments in a rapidly changing sporting environment (i.e., when interacting with opponents in team sports, etc.). Proprioceptive deficits could be implicated in a risk of injury, while it appears that various types of injury related to proprioceptive ability seem to occur at a higher rate in women [6,11]. There is literature indicating a higher rate of non-contact anterior cruciate ligament (ACL) injury in women as compared to their male counterparts [12], which is common in sports such as soccer, basketball, and volleyball [13], but also across other sports [14]. On the other hand, it has been proposed that the physiological changes associated with the menstrual cycle may be implicated in the observed gender differences. In women, proprioception has been suggested to be affected by the hormonal fluctuations and pain during menstruation [15]. Additionally, in physically active women with a moderate activity level (e.g. exercising two times per week), proprioception appeared to be worse when progesterone levels were higher (i.e. in the pre-menstrual phase) [16]. Also, changes in the sex hormones estradiol, progesterone, and testosterone appear to mediate changes in knee laxity across the menstrual cycle [17]. While there is a scarcity of data, the available literature overall points to a physiologically relevant effect of menstrual cycle phase on proprioception [6,15,16].

Fatigue appears to have a more established effect on the position sense and overall kinesthesia of both sexes. It has been shown that generalized fatigue, i.e., that induced by multi-joint activities, such as running, cycling, or walking, can affect neuromuscular performance (e.g., [8,18]). Moreover, localized muscle fatigue, induced by repetitive muscular voluntary contractions of specific muscle groups, has been shown to be sufficient to disturb postural control [19], which is another dimension of neuromuscular performance. In previous research, it has been observed that prolonged and extreme muscle strain reduces proprioception, motor control, and neuromuscular coordination [9,10]. It is also possible that the angle of a tested joint (and thus the length of the involved muscles) may affect proprioceptive ability (and propensity to injury), at least in passive movement (e.g., in drop and retention tests of the knee joint) [20]; however, not much is known.

Interestingly, the literature indicates that women may employ different movement patterns in fatigue [18,21]. For example, studies examining the effect of fatigue on lower extremity kinematics during landing from a jump have reported different compensatory kinematic and muscle activation patterns in women vs. men (e.g., increased gluteus maximus activation and decreased hip flexion in women) [21]. However, there is not much unequivocal information on how fatigue can affect healthy women’s proprioception, considering hormonal fluctuations, as most work has been conducted without controlling for the menstrual cycle of the participants [20] and/or was performed in women with a history of prior knee injury [22].

Thus, the present study aimed to examine whether localized muscle fatigue would affect knee proprioception (specifically, the accuracy of knee joint repositioning), and if any fatigue effect could be modulated by the phase of the menstrual cycle (follicular or luteal) and/or by the joint angle (using three target knee angles), in young physically active women without prior knee injury.

## 2. Materials and Methods

### 2.1. Subjects

The sample consisted of 20 active females (involved in team sports and/or contemporary dance) aged 19–30 years, with a regular monthly menstrual cycle (Table 1). The exclusion criteria were training age less than 3 years, lower extremity injury at least one year before the measurement, lower extremity pain, gynecological diseases, unstable menstrual cycle, or a cycle without changes in morning temperature (see below). This study was approved by the Internal Ethics Committee of the Department of PE and Sports Sciences (DPESS) at the University of Thessaly (code 3-1/12122012, in 2012), and the volunteers provided their written informed consent to participate in the study.

### 2.2. Study Design

The volunteers who responded to the study advertisement came to a first information meeting. If they met the basic criteria for participation and consented, they were given daily temperature recording forms. The forms were returned to the investigators after two months for evaluation. Then, the volunteers who met the criteria (see Section 2.4.1 and Section 2.4.2) were invited to participate. From the initial thirty responders, twenty met the participation criteria and continued to the experimental sessions, being randomly assigned to initiate their participation in the study either at their follicular or their luteal menstrual phase (in a switch replication design).

One week before the first experimental session, the morning, height and weight were recorded [23] and the dominant lower limb was identified (see below).

The two experimental sessions were performed in the morning (08:00–12:00) and within the same menstrual cycle (within 26 to 30 days depending on the subject’s characteristics) on two occasions. Before each experimental session, a blood sample was taken to confirm the phase of the cycle by determining hormone levels. During the experimental sessions, the participants performed an evaluation of knee position sense before and after the fatigue protocol in the dominant lower extremity (knee target angles being tested in a random order, i.e., while all were tested in all target knee joint angles, some were tested first at 30 degrees, others first at 45 degrees, and others first at 60 degrees).

### 2.3. Measuring Instruments

A Monark cycle ergometer (Vansbro, Sweden) was used to warm up. An isokinetic dynamometer (CYBEX NORM^®^, Ronkokoma, NY, USA) was used to determine the dominant lower extremity of the subjects. The same isokinetic dynamometer was used to assess knee position sense using a standardized protocol of knee proprioception [24]. The fatigue protocol (as in [25]) was also performed on the isokinetic dynamometer.

### 2.4. Preliminary Assessments

In preparation of the study, we evaluated the duration and regularity of the menstrual cycle and the subjects’ basic characteristics (body weight in kg and height in m were noted using scales and a stadiometer [SECA, Vogel & Halke, Hamburg, Germany]; body mass index (BMI) was calculated in kg/m^2^).

#### 2.4.1. Screening Period and Determination of Menstrual Cycle Phases

A body temperature recording diary form was used to record temperature fluctuations accompanying the menstrual cycle for two months to gauge the duration and regularity of menses [26], followed by blood tests to accurately determine estrogen (E2) and progesterone (P4) levels, at two phases (follicular–luteal) of the menstrual cycle. The temperature was recorded with a commercially available electronic thermometer with a resolution of 0.1 °C. Temperature was taken orally in the early morning immediately after waking up and before leaving the bed. The first day of the cycle was defined as the first day of menstruation and the last day was defined as the day before the start of the second menstrual cycle. Volunteers with an unstable menstrual cycle, incomplete forms, or no temperature changes were excluded from the study. Experimental session days were initially selected based on the temperature recording forms and later confirmed by blood analysis (see below).

Confirmation of the phase of the cycle was achieved by determining the levels of the hormones estradiol (E2) and progesterone (P4) in the blood by the ELISA method (1313, S/N: V12Ι 1210). Serum progesterone concentrations greater than 16 nmol/L confirmed ovulation. Serum P4 concentrations at 0.5–4.5 nmol/L and E2 at 18–147 pg/mL define the first half of the follicular phase (1–7 days), while serum P4 concentrations at 4.77–63.5 nmol/L and E2 at 43–214 pg/mL show the presence of the mid-luteal phase [27].

One experimental session was set to take place in the first half of the follicular phase, i.e., the 5th–6th day of the menstrual cycle (E2 at 18–147 pg/mL and P4 at 0.5–4.5 nmol/L). Another experimental session was set to take place on a day in the menstrual cycle that corresponded to the mid-luteal phase (E2 at 43–214 pg/mL and P4 at 4.77–63.5 nmol/L), which is defined as 7 days after ovulation (20th–23rd day of menstrual cycle) with a concomitant increase in serum progesterone (P4) [27]. The phase of the menstrual cycle in which the experimental part of this study started was random (i.e., some women were first tested at their follicular phase and others first tested at their luteal phase), in order to counterbalance the order of menstrual phase at first assessment.

#### 2.4.2. Determination of Dominant Lower Limb

The determination of the dominant lower extremity was based on the evaluation of maximum torque at both lower extremities with the isokinetic dynamometer (CYBEX NORM^®^, Ronkokoma, NY, USA), and was performed one week before the first experimental session. The test subjects arrived at the laboratory in the morning wearing sportswear. A 7 min warm up was performed on a Monark cycling ergometer (Vansbro, Sweden), at a speed of 70–80 rpm and 80-watt resistance followed by five minutes of guided stretching for the knee extensions and flexors. The determination of the dominant lower limb included 5 maximal repetitions of extension and flexion of the knee joint in a concentric manner at a constant angular velocity of 120°/s, according to a published protocol [25]. The anatomical zero (0°) was set at full knee extension. The range of motion was in total 110°, with movement allowed from 5° to 115°.

### 2.5. Experimental Sessions

Subjects returned to the laboratory on two occasions, corresponding to their follicular and luteal phases, for the assessment of knee position sense [7] in the rested and fatigue states. Body weight was recorded again in each visit.

#### 2.5.1. Position Sense Assessment

We set the experimental target angles at three knee angle positions (30°, 45°, 60°), with the knee at full extension being considered 0°. To avoid an order effect, the order of target replication varied between and within subjects.

Briefly, the procedure was as follows: each examinee was in a seated position (120° hip angle). The researcher lifted the lower limb (from its initial 90° knee flexion position), and placed it, in a random order, at the 30°, 45°, or 60° knee flexion for 10 s (while the subject did not actively participate in the procedure and did not see the knee movement), before returning the limb to 90° knee flexion. Each examinee was then asked to actively replicate the preceding angle. Once the subjects were satisfied with the angle they achieved, they held the limb in this position for 2 s. Three attempts were made for each angle, and data were recorded. A final result, as per the target angle, was calculated based on the average of three attempts.

Angle error (signed) was calculated as to the target angle, i.e., a participant reaching a knee angle of 28° when the target was 30° presented with a −2° angle error, while another participant reaching a knee angle of 32° when the target was 30° presented with a +2° angle error. Moreover, performance in recreating the target angle was calculated in terms of the percentage of target angle achieved (i.e., each target angle serving as 100%).

#### 2.5.2. Muscle Fatigue Protocol

The fatigue protocol involved continuous maximum repetitions of extension–flexion of the knee joint on the isokinetic dynamometer, in a concentric manner, at a constant angular velocity of 120°/s. The subjects performed maximal repetitions until the recorded torque, in three consecutive repetitions, decreased to 50% of their maximum torque [25], as measured a week earlier when determining the dominant lower limb.

### 2.6. Statistical Analysis

Power analysis was performed using the open-source software G*Power (3.1.9.2) and was used to calculate the minimum number of participants required to achieve reasonable power (>80%). A post hoc analysis revealed that in one of the main parameters related to the aims of the study (angle error at 60° target angle), we had enough power to detect statistically significant differences between the pre- and post-intervention comparisons (within groups: effect size dz = 1.3486974, total sample size = 20, t = −2.0930241, power (1-β err prob) = 0.9999102).

Data were checked for normal distribution using the Kolmogorov–Smirnov test. Descriptive statistics were performed and the values are presented as mean and standard deviation (Mean ± SD). A 3-way ANOVA analysis with repeated measures was performed. The first factor (variable) was muscle fatigue (two levels: before fatigue and after fatigue), the second factor was the phase of the menstrual cycle (two levels: follicular and luteal phase) and the third factor was the different target angles (30°, 45°, 60°). The relationship between target angle performance values was examined using a Pearson correlation coefficient.

Statistical significance was set at *p* < 0.05 and the Bonferroni post hoc test was applied. Moreover, effect size was reported as Partial Eta Squared (η_p_^2^), with values of 0.01 denoting a small effect, values 0.06–0.14 a medium effect, and values of >0.14 denoting a large effect.

## 3. Results

All 20 participants completed the experimental sessions, without missing data, and did not report any pain or discomfort. The collected data followed a normal distribution, and thus parametric statistics were used.

Participants’ age, weight, height, and calculated body mass index (BMI) are reported in Table 1. Body weight was stable, thus the data from the first visit are presented.

As described in the methods, we set three target angles for the participants’ proprioception testing and calculated the error as the degrees of deviation from the target. The results showed that the main effect of fatigue was significant, causing participants to produce numerically larger angular errors for all three target angles (F_(1,19)_ = 4,97, *p* = 0.038, η_p_^2^ = 0.207) when fatigued (Table 2).

No main effect of the menstrual cycle phase was found (F_(1,19)_ = 2.45, *p* > 0.05, η_p_^2^ = 0.114).

In addition, a main effect was found for the target angle (F_(2,18)_ = 9.93, *p* = 0.001, η_p_^2^ = 0.525), with more flexed target knee joint angles being associated with larger angular error deviations (in numerical terms). Specifically, the participants displayed increased angular errors when targeting 45° and 60° angles compared to the target angle of 30° (Table 2).

Overall, numerically larger angular error values were observed when targeting the 60° angle during the luteal phase of the cycle. This was true both before and after fatigue, with the error exceeding, in most cases, six degrees. In the follicular phase, performance was better in the rested state, while fatigue also caused errors exceeding six degrees. All angle errors had a negative sign (meaning more extended knee angles than intended) and are shown in Table 2.

The interaction between fatigue and target angle errors did not appear to modify the angular error and was not statistically significant (F_(2,18)_ = 2.39, *p* > 0.05, η_p_^2^ = 0.120). On the other hand, a tendency was observed for an interaction between fatigue and menstrual cycle phase (F_(1,19)_ = 3.56, *p* = 0.07, η_p_^2^ = 0.158). Additionally, the interaction between menstrual cycle phase and target angle was not statistically significant (F_(2,18)_ = 1,97, *p* = 0.168, η_p_^2^ = 0.180).

As an overall finding, there was not a significant interaction between all three factors: fatigue, menstrual cycle phase, and target angle errors (F_(2,18)_ = 0.92, *p* = 0.415, η_p_^2^ = 0.093).

When the performance was expressed as a percentage of the respective target angle, a main effect of fatigue was also found (F_(1,21)_ = 10.24, *p* < 0.05, η_p_^2^ = 0.328). Moreover, a main effect of the target angle was found (F_(2,20)_ = 7.76, *p* < 0.05, η_p_^2^ = 0.437), but no main effect of the menstrual phase was found (F_(1,21)_ = 1.75, *p* > 0.05, η_p_^2^ = 0.077).

**Figure 1:**
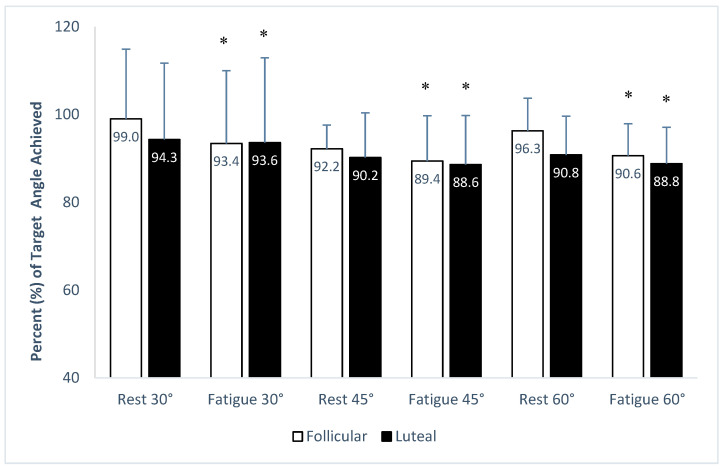
Performance of target angles expressed in percentage (%) of each respective target angle achieved in rest and fatigue conditions. White bars indicate the follicular and black bars indicate the luteal menstrual phases. Data (%) are shown as average values and standard deviation (mean ± SD). * significant differences (*p* < 0.05) from the respective rest condition.

A worse performance was again obtained at 60°, with average performance in the fatigued state in the luteal phase being as low as 89% of the intended target angle (Figure 1). Also, when examining performance at the 45° angle, we also found similar percentage deviations from the intended target angle (Figure 1).

Still, again, no statistically significant interactions were observed between fatigue and target angle (F_(2,20)_ = 1,26, *p* = 0.305, η_p_^2^ = 0.112), fatigue and menstrual cycle phase (F_(1,21)_ = 2.19, *p* = 0.153, η_p_^2^ = 0.095), or menstrual cycle phase and target angle (F_(2,20)_ = 0.83, *p* = 0.422, η_p_^2^ = 0.083).

As an overall finding, there was not a significant interaction between all three factors (variables): fatigue, menstrual cycle phase, and target angle (F_(2,20)_ = 0.53, *p* = 0.594, η_p_^2^ = 0.051).

Correlation analysis showed medium correlations (from r = 0.61 to r = 0.76, *p* < 0.05) between pre- and post-fatigue performance for all target angles in both menstrual phases (with pre–post-performance correlations becoming stronger, r > 0.70, in the luteal phase). However, rested performances at the follicular phase significantly correlated with rested performances at the luteal phase for the 30° (r = 0.68, *p* < 0.01) and 60° (r = 0.57, *p* < 0.01) target angles but not for the 45° target angle. Likewise, fatigued performances at the follicular phase significantly correlated with fatigued performances at the luteal phase for the 30° (r = 0,54, *p* < 0.05) and 60° (r = 0.59, *p* < 0.01) target angles but not for the 45° target angle.

From the correlations between the target angles, whether pre- or post-fatigue in the two menstrual cycle conditions, we noticed that all three angle performance values at the luteal phase correlated between them, with the weakest but significant correlations between the 30° and 60° target angles (e.g., in the fatigued state, r = 0.45, *p* < 0.05) and the strongest between the 45° and 60° target angles (e.g., in the rested state, r = 0.86, *p* < 0.01).

## 4. Discussion

The purpose of this study was to examine if the expected effect of localized muscle fatigue on knee position sense could be modulated by the phase of the menstrual cycle (follicular vs. luteal) and/or by the joint angle (using three target knee flexion angles: 30°, 45°, and 60°) in young physically active women. We found a consistent and significant effect of fatigue at all three target angles in both menstrual phases, with fatigued participants showing worse proprioceptive performance. The impact of fatigue on knee position sense seems to be independent of the menstrual phase. Notably, knee angle modulated performance, with increased angular errors recorded when targeting the 45° and 60° angles compared to the target angle of 30° in both the pre- and post-fatigue conditions. All angular errors had a negative sign (meaning more extended knee angles than intended).

Studies have investigated the role of fatigue in proprioception by including variations in the extent (general–local), type (isometric, isokinetic, isotonic), duration (continuous, repetitive), etc., of inducing fatigue. Fatigue has long been theorized to be a contributing factor in decreased proprioceptive acuity [28], and therefore a contributing factor to joint injury [11,14]. We found that localized muscular fatigue (using a protocol affecting both knee extensors and flexors) negatively and significantly affected knee extension proprioception during an open kinematic chain movement, especially when targeting the 45°and 60° knee angles. Our findings, in active women, partially agree with previous work on young active men using a similar fatigue protocol and active knee positioning by the participants [29], who noted a significant effect of fatigue on absolute, but not relative, error when employing a 30° knee target angle. However, another study in active men using a similar protocol reported differences in proprioception but only after general (treadmill running) fatigue and not after localized fatigue [8]. Still, in that latter study, the authors did not report the tested target angles, nor whether the order was random, and only a single attempt per target angle was performed. Considering that there is variability (positive and negative error) in such efforts, and, as noted in our results, greater accuracy in obtuse angles (more extended knee) vs. acute angles (more flexed knee), it is possible that the design of that study did not allow for a clear representation of knee repositioning error. In other studies using mixed-gender groups (e.g., Ju et al., 2010; [30]), the effects of fatigue on active movement repositioning of the knee angle (as relative error) were not statistically significant. That latter study, however, did not clearly report the target angles used, did not control for the menstrual phase, and only tested target angles once. However, they seem to also have noticed more extended knee reproductions following localized muscle fatigue [30], in agreement with our findings.

We did not study the effects of our fatigue protocol on knee flexion proprioception. In a study by Gear et al. (2011) [28] in a mixed-gender group of participants, also using a protocol affecting both knee extensors and flexors, a significant effect of localized fatigue on knee flexion proprioception was also found. In that latter study, it is noteworthy that both mild and intense (like ours) localized muscle fatigue significantly affected active joint repositioning.

We did not assess the effects of localized muscle fatigue on knee proprioception in a closed kinematic chain movement. However, in a study using cycling ergometry to induce fatigue (apparently submaximal exercise to exhaustion), and employing a weight-bearing movement (from standing to reproducing a target of 30° knee flexion), Changela et al. (2012) also reported a significant negative effect of generalized fatigue on knee flexion proprioception [31]. Thus, we cannot exclude that in our study the localized muscle fatigue protocol also influenced knee flexion performance in closed kinematic chain movements as well.

The literature indicates that fluctuations in gonadal hormones can have a neural effect. There is evidence that behavioral, neuromuscular, and neurological symptoms (incl. slower reflexes, etc.) appear during the luteal phase [15,16,32,33], but the exact neurohormonal mechanisms are not clear. Our study, employing an open kinematic chain active movement, did not find a statistically significant effect of the menstrual cycle in knee joint proprioception, whether at rest or after fatigue. But we noticed that the participants tended to show more accuracy in the follicular phase for all target angles than the luteal phase, but without those differences reaching statistical significance. In a Scandinavian study comparing healthy active women and female basketball players, it was found that knee joint proprioception indices were better during the ovulation phase [34]. A study by Aydog et al. (2005), also employing an open kinematic chain movement but in a semi-horizontal position, reported that resting knee proprioception was significantly reduced during active menstruation compared to the follicular and early luteal phases; importantly, they also found that accuracy was significantly better in the follicular vs. luteal phases for knee angles of 50° and 70° [35], in agreement with our overall, but not statistically significant, findings (for 45°and 60°, in our case). Other studies have shown no significant interaction between a ankle joint position sense test and the phases of the menstrual cycle [36].

It is not known if the fatigue protocol used presently might differentiate any possible effects of the menstrual cycle on active angle joint repositioning in a closed kinematic chain movement. In one of the few available studies, researchers employed a semi-squat movement from a standing position to a 30° knee angle at the rested state [37]. They reported that healthy female athletes presented with higher absolute values of reposition error during active menstruation; in that study, there were no significant differences in angle error between the mid-follicular and mid-luteal phases. It should be noted that in our study, while not statistically significant, both absolute and relative errors were numerically larger in the luteal phase vs. the follicular phase in both the resting and the fatigued state.

An issue of concern could be the possible effects of the menstrual phase on knee joint laxity that may predispose for injury. Still, Hertel et al. (2006) suggested that passive joint position sense (JPS) and joint laxity do not change across the menstrual cycle. They measured knee JPS with an isokinetic dynamometer in a seated position, employing passive movement [38]. Adachi et al. (2002) also suggested that ligament laxity does not affect the proprioceptive function of the knee, and that athletes may compensate with muscle contraction [39]. Also, Park et al. (2009) reported no significant changes in knee joint mechanics between the different phases of the menstrual cycle. Still, their findings indicated that an increase in knee joint laxity can be associated with internal adduction (valgus) and external rotation loads during a cutting maneuver and stopping from a jumping task [40]. We did not assess changes in knee laxity. However, none of our participants’ medical histories indicated knee laxity problems.

In the literature, studies examining kinaesthesia on passive motion indicate that the higher the level of general fitness, the better the ability to detect knee angle changes during passive motion. Specifically, Allison et al. (2016), recording passive motion detection, noted that physically active women with higher aerobic capacity and better fatigue resistance were able to better maintain their (passive) motion detection ability following a whole-body fatiguing battery of tests [41]. In another study examining knee extension proprioception during the passive movement of the tested limb, comparing professional dancers vs. generally active physical education students, both female and male, the researchers did not find an effect of fatigue on passive position sense in either group [18]. However, they reported an overall worse motion sense (i.e., awareness of passive movement initiation) by non-dancers after localized muscle fatigue vs. dancers. Unfortunately, that study did not control for the menstrual cycle, report the direction of error, or test for active repositioning error. They also completed all their testing within the same visit, perhaps obscuring any possible effects due to their study design. We cannot know what, if any, the effect of our fatigue protocol was on passive movement proprioception. Still, one could consider that perhaps professional dancers or elite athletes might have a heightened overall kinesthetic ability, at least for passive movement, compared to generally active or non-active women. To our knowledge, there is still no systematic study of overall proprioceptive abilities comparing generally active vs. elite athletes to reach safe conclusions.

Importantly, a main effect of the target angle on active knee position sense was found in the present study. More flexed target knee joint angles (i.e., more acute knee angles, such as 45 and 60°) were accompanied by statistically significantly larger angular error deviations compared to the extended target knee joint angle of 30°. This effect persisted in both the rested and the fatigued states (Table 2), and whether viewing data as degrees or % target values (Figure 1). The literature indicates, as discussed earlier, that decreased proprioception in the knee joint results in injuries, including anterior cruciate ligament (ACL) damage, with higher incidence rates in women. While the etiology is not clear, both biomechanical and physiological factors may be implicated, especially when athletes are fatigued. Studies examining the effect of fatigue on lower extremity kinematics during landing from a jump have reported different compensatory kinematic and muscle activation patterns in women vs. men, e.g., increased gluteus maximus activation and decreased hip flexion in women, especially after fatigue [21,42]. Decreased hip flexion is associated with greater knee extensor moments and risk of injury, as is observed in single-leg landings [43]. Moreover, sex-based differences have been observed in other body segments, e.g., men presenting greater trunk flexion after fatigue compared to women, perhaps as a mechanism that could minimize the increase in the tibiofemoral shear force resulting from the observed lower knee flexion after fatigue [44]. While we have focused on localized fatigue effects on the knee joint, our observations of persistent knee joint repositioning errors after fatigue in a population of healthy women, together with the above literature findings, point to the need for further research in safeguarding sport and occupational wellness for women whose professional roles necessitate operational resilience under fatigue, such as, for example, in the armed forces.

In the present study, only active knee extension repositioning was examined in an open kinematic chain protocol in a relatively homogenous group of active but non-elite sportswomen, because it was considered that active movements are more relevant to the real-world sport or occupational settings. Our study employed the criteria of a minimum of three years of physical training history and of no knee injuries, employing only young women of reproductive age with a stable menstrual cycle, to avoid any carrying effects of past knee injury, in contrast to some of the existing literature. Another advantage of our design is that the regularity of the menstrual cycle and the accompanying hormonal fluctuations were verified at a level of control not seen in other studies [45]. Moreover, by employing a switch replication approach, we addressed recently published recommendations on how best to perform experimental research in naturally cycling women considering that they differ greatly in their sensitivity to the cycle [45]. In the future, we should examine other parameters that can contribute to knee joint repositioning sense, including factors that are relevant to occupational roles, such as pressure or weight on the foot (e.g., equivalent to a ski boot or foot protective gear).

## 5. Conclusions

In conclusion, localized muscle fatigue can significantly reduce the accuracy of active knee joint repositioning in both the studied menstrual phases, luteal and follicular, in young, physically active healthy women. Notably, in the fatigue state, independently from the two menstrual cycle phases, active position sense performance presented with increased angular errors when targeting more acute (flexed) vs. more obtuse (extended) knee joint angles. The latter observation may have bearing for coaches and trainers, especially those handling athletes with knee injuries, or those in sports where extreme acute (bending) knee angles rapidly transition to obtuse (extending) knee angles, as in team sports where movement patterns can change rapidly in response to the opponent’s and/or the ball’s movement. Moreover, our observations can help towards setting injury prevention measures and improved planning of human resource deployment in physically demanding occupational settings.

## Figures and Tables

**Table 1:** Characteristics of the participants (*n* = 20).

	Minimum	Maximum	Mean	SD
Age (years)	19	30	23.80	3.17
Weight (kg)	51	76	60.22	6.31
Height (m)	1.49	1.83	1.64	0.85
BMI (kg/m^2^)	22.69	22.97	22.83	0.28

Data are expressed by range (min–max), mean, and standard deviation (±SD); BMI = body mass index.

**Table 2:** Deviation (in degrees) from target angles, before and after the fatigue protocol in the two examined phases of the menstrual cycle.

Target Angles	Menstrual Phases	Conditions	
		Rest	Fatigue
30°	Follicular	−0.34 ± 4.82	−2.00 ± 4.98 *
	Luteal	−2.08 ± 5.36	−0.597 ± 6.09 *
45°	Follicular	−3.57 ± 2.42 #	−4.76 ± 4.61 *#
	Luteal	−4.43 ± 4.59 #	−5.15 ± 5.03 *#
60°	Follicular	−2.24 ± 4.41 #	−5.65 ± 4.39 *#
	Luteal	−5.55 ± 5.29 #	−6.73 ± 4.99 *#

Data are expressed as mean ± SD (*n* = 20). * Significant differences *p* < 0.05 from rest; # significant differences compared to 30° target angle in either menstrual phase.

## Data Availability

The original contributions presented in the study are included in the article; further inquiries can be directed to the corresponding author.
